# Epidemiological Characteristics of Severe Fever with Thrombocytopenia Syndrome

**DOI:** 10.4269/ajtmh.24-0616

**Published:** 2025-02-18

**Authors:** Sakarn Charoensakulchai, Keita Matsuno, Emi E. Nakayama, Tatsuo Shioda, Hisham A. Imad

**Affiliations:** ^1^ Thai Travel Clinic, Hospital for Tropical Diseases, Faculty of Tropical Medicine, Mahidol University, Bangkok, Thailand;; ^2^ Division of Risk Analysis and Management, International Institute for Zoonosis Control, Hokkaido University, Sapporo, Japan;; ^3^ One Health Research Center, Hokkaido University, Sapporo, Japan;; ^4^ Institute for Vaccine Research and Development, HU-IVReD, Hokkaido University, Sapporo, Japan;; ^5^ International Collaboration Unit, International Institute for Zoonosis Control, Hokkaido University, Sapporo, Japan;; ^6^ Center for Infectious Disease Education and Research, Department of Viral Infections, Research Institute for Microbial Diseases, Osaka University, Osaka, Japan;; ^7^ Department of Clinical Tropical Medicine, Faculty of Tropical Medicine, Mahidol University, Bangkok, Thailand

## Abstract

Severe fever with thrombocytopenia syndrome (SFTS) is an emerging infectious disease primarily reported in Asia. This review aims to summarize studies on the epidemiological characteristics of SFTS. Literature from PubMed and Scopus was searched up to February 14, 2024. A total of 76 articles were eligible. Infections were reported in China, Japan, South Korea, and several other countries in Asia. The incidence of SFTS has been rising and reported from new areas across Asia. The incidence rate was highest in China, ranging from fewer than 0.1 to 4.2 cases per 100,000 population and reaching up to 127.6 cases per 100,000 population in some areas. Most cases occurred between April and December. Elderly farmers and veterinarians were the most affected group. Key epidemiological factors included direct contact with animals, outdoor work, vegetation near homes, rural or hilly residency, tick bites, and direct contact with blood or saliva from infected animals or humans.

## INTRODUCTION

Severe fever with thrombocytopenia syndrome (SFTS) is a tick-borne zoonotic infectious disease. The causative agent is a bandavirus (*Dabie bandavirus*), commonly known as severe fever with thrombocytopenia syndrome virus (SFTSV). This virus is also referred to as “*Huaiyangshan banyangvirus*,” “*SFTS bunyavirus*,” or “*SFTS phlebovirus*.” It belongs to the genus *Phlebovirus* within the family *Phenuiviridae* of the order *Buyavirales*.^1^ Since the first isolation and report of SFTSV in China in 2009, the virus has been documented in several other countries in Asia.^2^

Severe fever with thrombocytopenia syndrome virus is primarily transmitted to humans through the bite of the tick *Haemaphysalis longicornis*, which is widely distributed in East Asia. Transmission can also occur through contact with blood, saliva, nasal discharge, or urine from infected animals.^3^^–^^6^ Additionally, SFTSV can be found in various vertebrate reservoirs, including in mammals, such as carnivores, rodents, ungulates, and insectivores, and in birds.^4^^,^^5^^,^^7^

The clinical presentation of SFTS often resembles an acute undifferentiated febrile illness, with signs and symptoms overlapping with other tropical diseases, such as rickettsiosis.^8^ This overlap can delay diagnosis and treatment, increasing the risk of mortality. Despite the availability of an antiviral proven to be effective against SFTSV infection, awareness of the disease remains limited, primarily because of its recent discovery and the relative low number of documented cases. With its high case fatality rates (CFRs) from delayed treatment in high-risk groups, broad host range, and wide geographic distribution, SFTS is an emerging viral disease that poses an alarming public health risk. Increased awareness and information about the disease are essential for effective prevention and management. This review focuses on epidemiological characteristics of SFTSV infection in humans.

## SEARCH STRATEGY

We accessed articles published until February 14, 2024 through PubMed and Scopus. We included research articles that focused on the epidemiology and characteristics of SFTSV infection. Exclusion was applied to review articles, correspondences, perspectives, letters, book chapters, nonhuman studies, and non-English publications. No formal review protocol was registered for this review. The search strategy is shown in Supplemental Table 1.

## RESULTS

**Figure 1.**
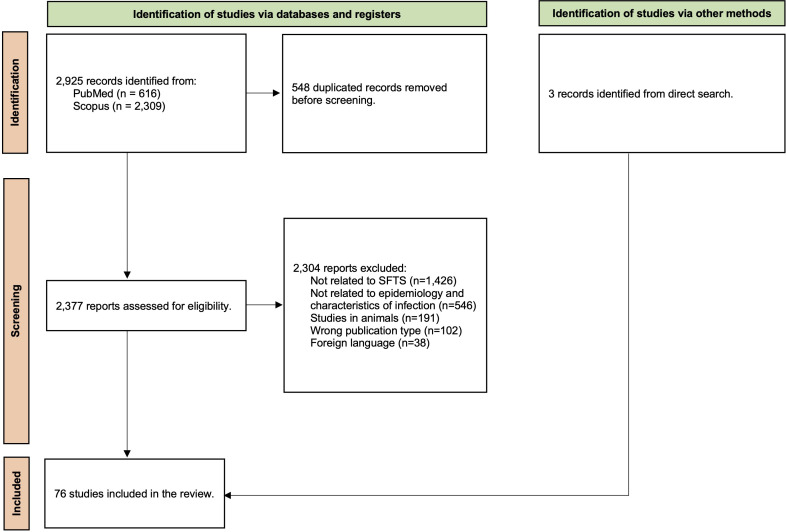
Screening process. SFTS = severe fever with thrombocytopenia syndrome.

A total of 2,925 articles were initially identified. After removing duplicates and excluded articles, 73 articles met the inclusion criteria. Additionally, 3 more articles were identified through direct search, bringing the total to 76 articles for this review. The screening process is shown in Figure 1. Supplemental Table 2 provides a summary of all included studies.

### Epidemiology of SFTS.

**Figure 2.**
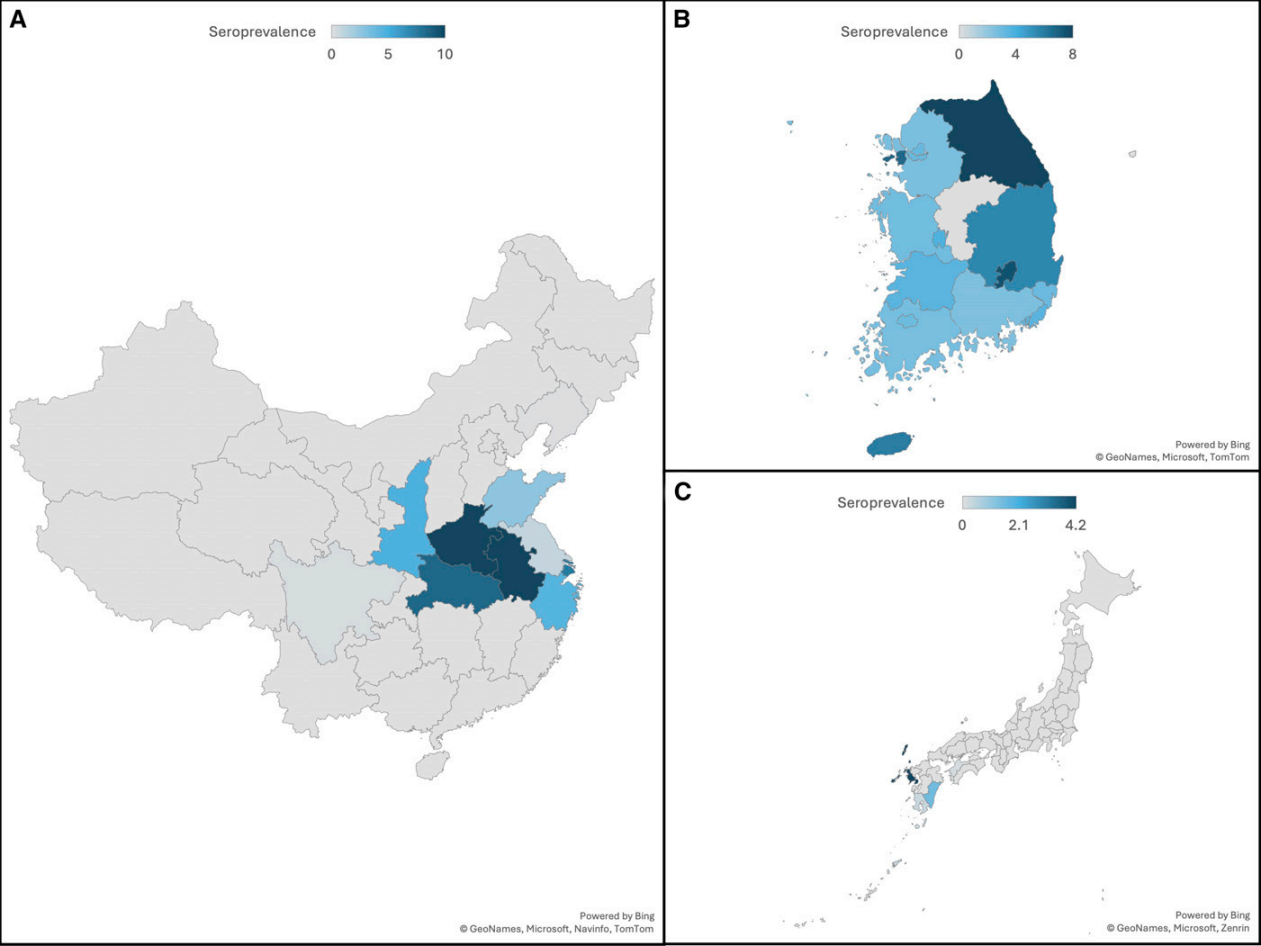
Epidemiological maps of severe fever with thrombocytopenia syndrome. (**A**) China. (**B**) South Korea. (**C**) Japan.

Severe fever with thrombocytopenia syndrome is endemic to East Asia, including China, South Korea, and Japan.^9^^–^^11^ Although the first case of SFTS was described in 2009, several clusters of patients with symptoms like those of SFTS in China (most were clinically diagnosed with human anaplasmosis) were retrospectively tested positive for SFTSV antibody and RNA.^12^^,^^13^ Thus, the disease may have been emerging in these areas long before the identification of SFTS. Following the emergence of SFTS in 2009, it was rapidly reported to neighboring provinces in China, South Korea, and Japan, with infections also reported in Myanmar, Thailand, Vietnam, and Pakistan.^10^^,^^14^^–^^17^ Figure 2 illustrates the epidemiological map of SFTS.

### East Asia.

#### China.

The distribution of SFTS in China is primarily concentrated in the central and eastern regions of the country.^10^^,^^18^^,^^19^ Patterns of SFTS distribution in China have been observed to occur in clusters, particularly in provinces such as Jiangsu, Liaoning, Shandong, Zhejiang, Anhui, Henan, and Hubei.^10^^,^^13^^,^^20^^–^^23^ These clusters often share similar hilly and mountainous landscapes.^10^ Between 2010 and 2019, China reported a total of 13,824 SFTS cases, with 64.4% of them being laboratory confirmed.^23^ The seroprevalence of SFTSV in China ranges from 0.3% to 20.2%, with many infections remained undiagnosed or asymptomatic.^12^^,^^24^^–^^40^ The incidence of SFTS has significantly increased, and its distribution areas have expanded over the years.^10^^,^^18^^,^^23^^,^^41^^–^^45^ The average incidence of SFTS varies greatly across different regions, ranging from less than 0.1 to 4.2 cases per 100,000 population.^10^^,^^18^^,^^20^^,^^41^^,^^44^^–^^51^ In certain areas, the incidence can be exceptionally high, reaching up to 127.6 cases per 100,000 population.^43^ Although the disease is predominantly found in hilly and mountainous areas, the highest incidences and seroprevalences have been reported in coastal and island regions, such as the Dachen Islands off the coast of Zhejiang.^27^^,^^33^^,^^43^ The seroprevalence of SFTSV on Dachen Islands was approximately 3.0–4.8%, exceeding that of several mainland areas.^27^^,^^33^

#### South Korea.

The seroprevalence of SFTSV in humans in South Korea ranged from 0.2% to 5.9%.^11^^,^^52^^–^^57^ The seroprevalence was higher on islands than on the mainland as evidenced by the rates on Jeju Island (2.4%) and Mui Island (5.9%).^56^^,^^57^ Among veterinary hospital staff, the seroprevalence ranged from approximately 1.9% to 3.9%.^58^ The overall incidence of SFTS was 0.1 cases per 100,000 population, with most cases reported in the eastern and southern regions, including Jeju Island.^59^^,^^60^ In the northeastern province of Gangwon, a highly prevalent region, the incidence ranged from 0.3 to 8.0 per 100,000 population.^60^^,^^61^ On Jeju Island, the incidence was sometimes reported to be the highest in South Korea, reaching 1.3 cases per 100,000 population.^60^ This high incidence on Jeju Island can be attributed to the predominantly agricultural occupations of the island’s inhabitants as well as the wide range of farmlands and natural habitats suitable for tick habitation.^57^

#### Japan.

Seroprevalence of SFTSV in Japan ranged from 0.0% to 4.2%.^9^^,^^62^^–^^67^ Samples from donated blood showed seroprevalence rates as low as 0.0%, indicating a low rate of infection among the general population.^63^^,^^66^ However, higher seroprevalence rates were found in veterinarian personnel, ranging from 2.2% to 4.2%.^65^^,^^66^ The incidence of SFTS in Japan was fewer than 0.1 cases per 100,000 population.^68^^,^^69^ Cases were predominantly reported from the western part of Japan, attributed to its mountainous areas.^63^^,^^68^

### Other regions in Asia.

Severe fever with thrombocytopenia syndrome virus infections have also been reported in various regions outside the Far East. In Myanmar, studies conducted in two cities revealed that 3.3% of patients suspected of having rickettsiosis were infected with SFTS.^14^ In Vietnam, seroprevalence studies showed that 3.6% of residents showed evidence of past exposure to SFTSV.^15^ Additionally, sporadic reports of SFTSV infection have also been reported in Thailand, where affected patients lived in urban and suburban areas and had no history of travel to forested area before the onset of symptoms.^16^ Reports from Pakistan indicate a wide range of seroprevalence among farmers: from 2.5% to 46.7%.^17^

### Epidemiological characteristics of SFTSV infection.

Severe fever with thrombocytopenia syndrome cases in endemic areas are typically heightened during April and December.^10^^,^^13^^,^^19^^,^^20^^,^^22^^,^^23^^,^^41^^,^^42^^,^^50^^,^^59^^,^^60^^,^^68^^–^^75^ Severe fever with thrombocytopenia syndrome virus seropositivity rates are higher in rural areas compared with urban settings.^52^ The majority of confirmed SFTS cases are farmers, with seroprevalence rates among farmers higher than in the general population because of more frequent exposure to ticks.^12^^,^^13^^,^^18^^,^^20^^,^^23^^,^^31^^,^^42^^,^^45^^,^^46^^,^^53^^,^^67^^,^^68^^,^^71^^,^^74^^–^^78^ Agricultural activities as well as longer working hours on farmlands are associated with SFTSV seropositivity.^34^

As a tick-borne disease, environmental exposures that increase the risk of tick bites—such as engaging in outdoor activities and working or residing in areas with vegetation, such as forested regions, tea plantations, and hilly areas—pose risks for SFTSV infection.^13^^,^^21^^,^^24^^,^^31^^,^^38^^,^^39^^,^^43^^,^^46^^,^^68^^–^^70^^,^^72^^,^^76^^–^^79^ The presence of animals that are susceptible for SFTSV infection, such as rats and cats, as well as high densities of goats and cattle in living areas further increase the risk of SFTSV infection.^13^^,^^18^^,^^21^^,^^25^^,^^26^^,^^34^^,^^38^^,^^41^^,^^72^^,^^77^^,^^79^ The seroprevalences observed in wild and domesticated animals were 45.7–66.8% in goats, 13.2–36.7% in cattle, 33.1% in cats, 7.4–29.5% in dogs, 25.0% in wild boar, 25.0% in deer, 3.2–4.7% in pigs, 3.2–4.4% in rodents, 2.7% in hedgehogs, 1.7% in geese, and 1.2–9.6% in chickens.^8^^,^^9^^,^^29^^,^^34^^,^^65^ In coastal areas of China, living on islands is associated with SFTSV seropositivity and higher incidence rates.^33^^,^^43^ This high incidence on islands may be because of the dense forest environments, such as on Dachen Island, which provide a suitable habitat for ticks and intensify the transmission of SFTSV to animals and humans.^33^^,^^43^

**Figure 3.**
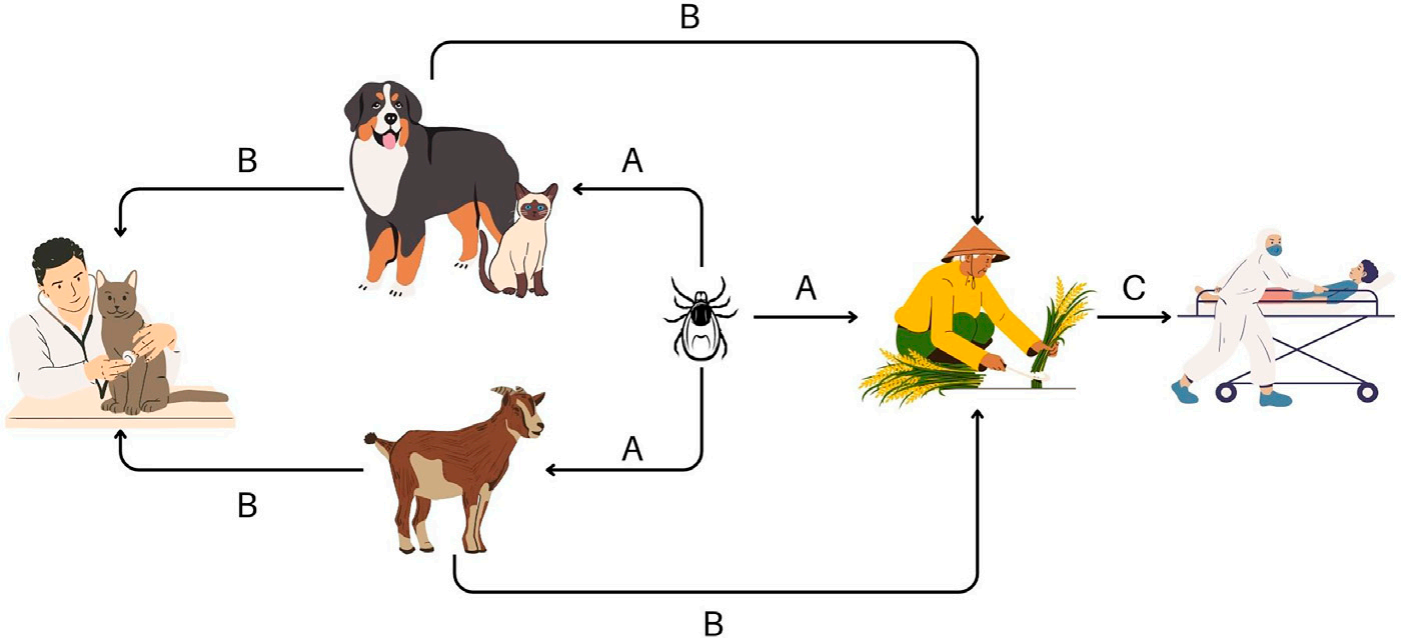
Transmission modes of severe fever with thrombocytopenia syndrome virus. (**A**) Tick bite. (**B**) Contact with infected animal’s blood and body fluid. (**C**) Contact with patient’s blood and body fluid.

Direct contact with animals and livestock is associated with SFTSV infection, with some infected people, particularly among veterinarian personnel, having directly contacted the body fluids of cats and dogs.^31^^,^^69^ Person-to-person transmission of SFTSV is possible through direct contact with infected patients’ body fluids, such as blood, or through exposure to corpses.^7^^,^^19^^,^^22^^,^^80^ Face-to-face exposures within 50 cm or exposure times of 30 minutes or longer with the patient increase the risk of transmission.^81^ Seroprevalence rates are higher among family members or individuals living in the same household as the index patient compared with those who did not have such close contact.^19^^,^^25^^,^^82^ The transmission modes of SFTSV are shown in Figure 3.

The incidence and fatality rates of SFTS typically increase with age, with most cases occurring in elderly individuals, likely reflecting the distribution of elderly populations in rural areas.^12^^,^^13^^,^^17^^–^^21^^,^^23^^,^^27^^,^^38^^,^^41^^,^^42^^,^^45^^,^^46^^,^^51^^–^^53^^,^^56^^,^^59^^,^^67^^,^^68^^,^^70^^,^^71^^,^^73^^,^^74^ The incidence rate in individuals over 40 years old is notably higher, suggesting that older individuals are more susceptible to severe clinical symptoms requiring medical attention compared with younger individuals.^51^ However, the seroprevalence of SFTSV does not differ significantly by age group, indicating that the higher incidence in elderly individuals may be attributed to the more severe clinical symptoms experienced by this demographic.^51^ Despite increasing incidence, the CFRs have been decreasing in recent years in China and South Korea.^23^^,^^31^^,^^41^^,^^59^^,^^61^^,^^71^^,^^74^^,^^83^ This decline in CFRs is attributed to several factors, including the expansion of surveyed populations from only hospitalized patients to nationwide populations in recent years along with improved diagnostic methods and an increase in the use of plasma exchange and ribavirin treatments.^59^^,^^83^^,^^84^ These advancements also enhance the ability to recognize early-stage infections, contributing to better management and outcomes.^84^

Gender may also play a role in SFTS risk, with some studies indicating a higher prevalence in females compared with males, possibly because of factors such as increased tick exposure during agricultural activities, heightened susceptibility to developing the disease after infection, or reporting biases owing to more frequent doctor visits after symptom onset.^10^^,^^17^^,^^23^^,^^41^^,^^53^^,^^83^ Conversely, some studies have suggested that male gender is associated with SFTS because of longer periods spent in farming areas, which subsequently heighten the risk of tick exposure.^34^^,^^48^ Variations in sample composition and the geographic regions from which data were collected may account for these differences.^23^ For instance, a study in Liaoning, China had a higher proportion of male than female samples,^48^ whereas in Henan, China, more females were involved in tea farming, which may influence the gender ratio of SFTSV infections in that area.^8^^,^^10^ Characteristics of SFTSV infection are presented in Table 1.

**Table 1:** Characteristics of SFTSV infection

Type of Case/Population	Risk Factors of SFTS
Farmers	Elderly people Occurred during April to December Working or spending extensive time outdoor Living in hilly area Living in rural setting Presence of reservoir hosts in the house Presence of vegetation in living and working areas Raising domesticated animals near living areas and having direct contact with domesticated animals Had history of tick bite
Veterinary staffs	Had direct contact with body fluids of symptomatic cats and dogs Did not properly wear a full personal protective equipment while performing a procedure
Secondary SFTS cases	Being in the same household of index SFTS cases Being health care provider providing procedures for SFTS patients with secretion or blood dispersal Did not properly wear full personal protective equipment while performing a procedure Had direct contact with blood and body fluid

SFTS = severe fever with thrombocytopenia syndrome.

## CONCLUSION

The incidence of SFTS shows an increasing trend and the spread to wider geographic areas. High incidence rates are observed in rural and island regions, particularly in China and South Korea. Most infected people are elderly farmers who are frequently exposed to ticks through contact with domesticated animals or vegetations. The virus can spread through the saliva, nasal discharge, urine, and blood of infected animals and humans, putting veterinarians, health care workers, and close contacts at risk. A One Health approach, including surveillance of both humans and animals in endemic regions and raising awareness about preventive measures, is important.

## Supplemental Materials

10.4269/ajtmh.24-0616 Supplemental Materials

## References

[1] Abudurexiti A , 2019. Taxonomy of the order Bunyavirales: Update 2019. Arch Virol 164: 1949–1965. 31065850 10.1007/s00705-019-04253-6 PMC6641860

[2] Sharma D Kamthania M, 2021. A new emerging pandemic of severe fever with thrombocytopenia syndrome (SFTS). Virusdisease 32: 220–227. 33942022 10.1007/s13337-021-00656-9 PMC8082055

[3] Yu X-J , 2011. Fever with thrombocytopenia associated with a novel bunyavirus in China. N Engl J Med 364: 1523–1532. 21410387 10.1056/NEJMoa1010095 PMC3113718

[4] Seo JW Kim D Yun N Kim DM, 2021. Clinical update of severe fever with thrombocytopenia syndrome. Viruses 13: 1213. 34201811 10.3390/v13071213 PMC8310018

[5] Casel MA Park SJ Choi YK, 2021. Severe fever with thrombocytopenia syndrome virus: Emerging novel phlebovirus and their control strategy. Exp Mol Med 53: 713–722. 33953322 10.1038/s12276-021-00610-1 PMC8178303

[6] Ye C Qi R, 2021. Risk factors for person-to-person transmission of severe fever with thrombocytopenia syndrome. Infect Control Hosp Epidemiol 42: 582–585. 33161921 10.1017/ice.2020.1258

[7] Chen C Li P Li KF Wang HL Dai YX Cheng X Yan JB, 2019. Animals as amplification hosts in the spread of severe fever with thrombocytopenia syndrome virus: A systematic review and meta-analysis. Int J Infect Dis 79: 77–84. 30500443 10.1016/j.ijid.2018.11.017

[8] Li JC Zhao J Li H Fang LQ Liu W, 2022. Epidemiology, clinical characteristics, and treatment of severe fever with thrombocytopenia syndrome. Infect Med (Beijing) 1: 40–49. 38074982 10.1016/j.imj.2021.10.001 PMC10699716

[9] Kimura T , 2018. Seroprevalence of severe fever with thrombocytopenia syndrome (SFTS) virus antibodies in humans and animals in Ehime prefecture, Japan, an endemic region of SFTS. J Infect Chemother 24: 802–806. 30017796 10.1016/j.jiac.2018.06.007

[10] Miao D , 2021. Epidemiology and ecology of severe fever with thrombocytopenia syndrome in China, 2010–2018. Clin Infect Dis 73: e3851–e3858. 33068430 10.1093/cid/ciaa1561 PMC8664468

[11] Kim CM Han MA Yun NR Bang MS Lee YM Lee B Kim DM, 2023. Seroprevalence of severe fever with thrombocytopenia syndrome using specimens from the Korea National Health & Nutrition Examination Survey. PLoS Negl Trop Dis 17: e0011097. 36947741 10.1371/journal.pntd.0011097 PMC10032665

[12] Cui F Cao HX Wang L Zhang SF Ding SJ Yu XJ Yu H, 2013. Clinical and epidemiological study on severe fever with thrombocytopenia syndrome in Yiyuan County, Shandong Province, China. Am J Trop Med Hyg 88: 510–512. 23339197 10.4269/ajtmh.11-0760 PMC3592533

[13] Hou S Zhang N Liu J Li H Liu X Liu T, 2023. Epidemiological characteristics and risk factors of severe fever with thrombocytopenia syndrome in Yantai City, Shandong Province. Open Forum Infect Dis 10: ofad141. 37065987 10.1093/ofid/ofad141 PMC10096902

[14] Win AM , 2020. Genotypic heterogeneity of *Orientia tsutsugamushi* in scrub typhus patients and thrombocytopenia syndrome co-infection, Myanmar. Emerg Infect Dis 26: 1878–1881. 32687023 10.3201/eid2608.200135 PMC7392420

[15] Tran XC , 2022. Serological evidence of severe fever with thrombocytopenia syndrome virus and IgM positivity were identified in healthy residents in Vietnam. Viruses 14: 2280. 36298836 10.3390/v14102280 PMC9607213

[16] Rattanakomol P Khongwichit S Linsuwanon P Lee KH Vongpunsawad S Poovorawan Y, 2022. Severe fever with thrombocytopenia syndrome virus infection, Thailand, 2019–2020. Emerg Infect Dis 28: 2572–2574. 36418010 10.3201/eid2812.221183 PMC9707585

[17] Zohaib A , 2020. Serologic evidence of severe fever with thrombocytopenia syndrome virus and related viruses in Pakistan. Emerg Infect Dis 26: 1513–1516. 32568060 10.3201/eid2607.190611 PMC7323538

[18] Liu K , 2015. A national assessment of the epidemiology of severe fever with thrombocytopenia syndrome, China. Sci Rep 5: 9679. 25902910 10.1038/srep09679 PMC4407178

[19] Fang X Hu J Peng Z Dai Q Liu W Liang S Li Z Zhang N Bao C, 2021. Epidemiological and clinical characteristics of severe fever with thrombocytopenia syndrome bunyavirus human-to-human transmission. PLoS Negl Trop Dis 15: e0009037. 33930022 10.1371/journal.pntd.0009037 PMC8087050

[20] Liang S Li Z Zhang N Wang X Qin Y Xie W Bao C Hu J, 2023. Epidemiological and spatiotemporal analysis of severe fever with thrombocytopenia syndrome in eastern China, 2011–2021. BMC Public Health 23: 508. 36927782 10.1186/s12889-023-15379-3 PMC10019416

[21] Liu W Dai K Wang T Zhang H Wu J Liu W Fang L, 2023. Severe fever with thrombocytopenia syndrome incidence could be associated with ecotone between forest and cultivated land in rural settings of central China. Ticks Tick Borne Dis 14: 102085. 36435169 10.1016/j.ttbdis.2022.102085

[22] Chen Q Yang D Zhang Y Zhu M Chen N Yushan Z, 2022. Transmission and mortality risk assessment of severe fever with thrombocytopenia syndrome in China: Results from 11-years’ study. Infect Dis Poverty 11: 93. 36058928 10.1186/s40249-022-01017-4 PMC9440863

[23] Huang X Li J Li A Wang S Li D, 2021. Epidemiological characteristics of severe fever with thrombocytopenia syndrome from 2010 to 2019 in mainland China. Int J Environ Res Public Health 18: 3092. 33802869 10.3390/ijerph18063092 PMC8002760

[24] Lyu Y , 2016. Seroprevalence and risk factors of severe fever with thrombocytopenia syndrome virus infection in endemic areas. Infect Dis (Lond) 48: 544–549. 27117875 10.3109/23744235.2016.1165351

[25] Sun JM , 2015. Seroprevalence of severe fever with thrombocytopenia syndrome virus in southeastern China and analysis of risk factors. Epidemiol Infect 143: 851–856. 24866248 10.1017/S0950268814001319 PMC4411641

[26] Liang S , 2014. Seroprevalence and risk factors for severe fever with thrombocytopenia syndrome virus infection in Jiangsu Province, China, 2011. Am J Trop Med Hyg 90: 256–259. 24343883 10.4269/ajtmh.13-0423 PMC3919226

[27] Shen W Lin H Weng J Hu Y Liu Y Li J Xu F Guan C Sun J, 2019. Seroprevalence of severe fever with thrombocytopenia syndrome virus antibodies among inhabitants of Dachen Island, eastern China. Ticks Tick Borne Dis 10: 647–650. 30826250 10.1016/j.ttbdis.2019.02.009

[28] Huang X Zhang Z Jin G Wang X Tan C Yin H Wang S, 2017. Presence of antibodies against severe fever with thrombocytopenia syndrome virus in non-endemic areas of China. Jpn J Infect Dis 70: 248–251. 27580581 10.7883/yoken.JJID.2016.214

[29] Tian H , 2017. Severe fever with thrombocytopenia syndrome virus in humans, domesticated animals, ticks, and mosquitoes, Shaanxi Province, China. Am J Trop Med Hyg 96: 1346–1349. 28719252 10.4269/ajtmh.16-0333 PMC5462569

[30] Li D Shao L Bi Y Niu G, 2018. Neutralizing antibodies to severe fever with thrombocytopenia syndrome virus in general population, Shandong Province, China. Sci Rep 8: 15401. 30337627 10.1038/s41598-018-33884-z PMC6193936

[31] You E Wang L Zhang L Wu J Zhao K Huang F, 2021. Epidemiological characteristics of severe fever with thrombocytopenia syndrome in Hefei of Anhui Province: A population-based surveillance study from 2011 to 2018. Eur J Clin Microbiol Infect Dis 40: 929–939. 33188497 10.1007/s10096-020-04098-x

[32] Du Y Cheng N Li Y Wang H You A Su J Nie Y Ma H Xu B Huang X, 2019. Seroprevalance of antibodies specific for severe fever with thrombocytopenia syndrome virus and the discovery of asymptomatic infections in Henan Province, China. PLoS Negl Trop Dis 13: e0007242. 31765376 10.1371/journal.pntd.0007242 PMC6901261

[33] Zu Z Lin H Hu Y Zheng X Chen C Zhao Y Zhang Z He N, 2024. Seroprevalence and transmission of severe fever with thrombocytopenia syndrome virus in a coastal endemic area in southeastern China. Ticks Tick Borne Dis 15: 102277. 37981467 10.1016/j.ttbdis.2023.102277

[34] Li Z , 2014. Seroprevalence of antibodies against SFTS virus infection in farmers and animals, Jiangsu, China. J Clin Virol 60: 185–189. 24793967 10.1016/j.jcv.2014.03.020

[35] Zeng P , International Component of the NHLBI Recipient Epidemiology and Donor Evaluation Study-III (REDS-III), 2015. A study of seroprevalence and rates of asymptomatic viremia of severe fever with thrombocytopenia syndrome virus among Chinese blood donors. Transfusion 55: 965–971. 25496479 10.1111/trf.12953 PMC4428969

[36] Zhang L , 2014. Antibodies against severe fever with thrombocytopenia syndrome virus in healthy persons, China, 2013. Emerg Infect Dis 20: 1355–1357. 25061813 10.3201/eid2008.131796 PMC4111193

[37] Hu C , 2015. The severe fever with thrombocytopenia syndrome bunyavirus (SFTSV) antibody in a highly endemic region from 2011 to 2013: A comparative serological study. Am J Trop Med Hyg 92: 479–481. 25624404 10.4269/ajtmh.14-0447 PMC4350533

[38] Ye XL , 2021. Infection with severe fever with thrombocytopenia virus in healthy population: A cohort study in a high endemic region, China. Infect Dis Poverty 10: 133. 34794512 10.1186/s40249-021-00918-0 PMC8600349

[39] Xing X , 2017. A case-control study of risk sources for severe fever with thrombocytopenia syndrome in Hubei Province, China. Int J Infect Dis 55: 86–91. 28088586 10.1016/j.ijid.2017.01.003

[40] Xing X , 2016. Natural transmission model for severe fever with thrombocytopenia syndrome bunyavirus in villages of Hubei Province, China. *Medicine (Baltimore)* 95: e2533. 26825892 10.1097/MD.0000000000002533 PMC5291562

[41] Wang T Li XL Liu M Song XJ Zhang H Wang YB Tian BP Xing XS Li SY, 2017. Epidemiological characteristics and environmental risk factors of severe fever with thrombocytopenia syndrome in Hubei Province, China, from 2011 to 2016. Front Microbiol 8: 387. 28337190 10.3389/fmicb.2017.00387 PMC5340758

[42] Sun J Lu L Wu H Yang J Ren J Liu Q, 2017. The changing epidemiological characteristics of severe fever with thrombocytopenia syndrome in China, 2011–2016. Sci Rep 7: 9236. 28835633 10.1038/s41598-017-08042-6 PMC5569157

[43] Zu Z Hu Y Zheng X Chen C Zhao Y Jin Y Lin H He N, 2022. A ten-year assessment of the epidemiological features and fatal risk factors of hospitalised severe fever with thrombocytopenia syndrome in eastern China. Epidemiol Infect 150: e131. 35726737 10.1017/S0950268822001108 PMC9306006

[44] Zhang Q Liu W Wang W Zhang L Li J Tang R Jin J Chen W Zhang L, 2023. Analysis of spatial-temporal distribution characteristics and natural infection status of SFTS cases in Hefei from 2015 to 2021. Environ Health Prev Med 28: 70. 37967947 10.1265/ehpm.23-00149 PMC10654213

[45] Chen R Kou Z Xu L Cao J Liu Z Wen X Wang Z Wen H, 2019. Analysis of epidemiological characteristics of four natural-focal diseases in Shandong Province, China in 2009–2017: A descriptive analysis. PLoS One 14: e0221677. 31454372 10.1371/journal.pone.0221677 PMC6711524

[46] Liu K , 2014. Epidemiologic features and environmental risk factors of severe fever with thrombocytopenia syndrome, Xinyang, China. PLoS Negl Trop Dis 8: e2820. 24810269 10.1371/journal.pntd.0002820 PMC4014392

[47] Wang Y Pang B Ma W Kou Z Wen H, 2022. Spatiotemporal analysis of severe fever with thrombocytopenia syndrome in Shandong Province, China, 2014–2018. BMC Public Health 22: 1998. 36319995 10.1186/s12889-022-14373-5 PMC9624039

[48] Wang Z , 2022. Epidemiological characteristics of severe fever with thrombocytopenia syndrome and its relationship with meteorological factors in Liaoning Province, China. Parasit Vectors 15: 283. 35933453 10.1186/s13071-022-05395-4 PMC9357322

[49] Wang Y Pang B Ma W Kou Z Wen H, 2022. Analysis of the spatial-temporal components driving transmission of the severe fever with thrombocytopenia syndrome in Shandong Province, China, 2016–2018. Transbound Emerg Dis 69: 3761–3770. 36265799 10.1111/tbed.14745

[50] Ding FY , 2023. Projecting spatiotemporal dynamics of severe fever with thrombocytopenia syndrome in the mainland of China. Glob Chang Biol 29: 6647–6660. 37846616 10.1111/gcb.16969

[51] Ding S , 2014. Age is a critical risk factor for severe fever with thrombocytopenia syndrome. PLoS One 9: e111736. 25369237 10.1371/journal.pone.0111736 PMC4219771

[52] Kim KH Ko MK Kim N Kim HH Yi J, 2017. Seroprevalence of severe fever with thrombocytopenia syndrome in southeastern Korea, 2015. J Korean Med Sci 32: 29–32. 27914128 10.3346/jkms.2017.32.1.29 PMC5143294

[53] Han MA Kim CM Kim DM Yun NR Park SW Han MG Lee WJ, 2018. Seroprevalence of severe fever with thrombocytopenia syndrome virus antibodies in rural areas, South Korea. Emerg Infect Dis 24: 872–874. 29664384 10.3201/eid2405.152104 PMC5938763

[54] Kim YR Yun Y Bae SG Park D Kim S Lee JM Cho NH Kim YS Lee KH, 2018. Severe fever with thrombocytopenia syndrome virus infection, South Korea, 2010. Emerg Infect Dis 24: 2103–2105. 30334706 10.3201/eid2411.170756 PMC6199997

[55] Noh JY Song JY Bae JY Park MS Yoon JG Cheong HJ Kim WJ, 2021. Seroepidemiologic survey of emerging vector-borne infections in South Korean forest/field workers. PLoS Negl Trop Dis 15: e0009687. 34407077 10.1371/journal.pntd.0009687 PMC8405005

[56] Kim-Jeon MD Moon SM Oh SS Kim H Koh Y-J Jegal S Han SY Lee MY Gong YW Park YS, 2022. Seroprevalence of severe fever with thrombocytopenia syndrome virus in Mui Island, rural area, Incheon, South Korea. J Bacteriol Virol 52: 64–71.

[57] Yoo JR Heo ST Kim M Song SW Boo JW Lee KH, 2019. Seroprevalence of severe fever with thrombocytopenia syndrome in the agricultural population of Jeju Island, Korea, 2015–2017. Infect Chemother 51: 337–344. 31668024 10.3947/ic.2019.51.4.337 PMC6940373

[58] Kim CM Kim DM Bang MS Seo JW Yun NR Kim DY Han MA Hwang JH Park SK, 2023. The seroprevalence of severe fever with thrombocytopenia syndrome: An epidemiological study of Korean veterinary hospital workers. Viruses 15: 609. 36992318 10.3390/v15030609 PMC10052674

[59] Park SW Ryou J Choi WY Han MG Lee WJ, 2016. Epidemiological and clinical features of severe fever with thrombocytopenia syndrome during an outbreak in South Korea, 2013–2015. Am J Trop Med Hyg 95: 1358–1361. 27928084 10.4269/ajtmh.16-0251 PMC5154450

[60] Choi SJ ; Korea SFTS Clinical Network, 2016. Severe fever with thrombocytopenia syndrome in South Korea, 2013–2015. PLoS Negl Trop Dis 10: e0005264. 28033338 10.1371/journal.pntd.0005264 PMC5226827

[61] Moon MY , 2023. Genetic diversity, regional distribution, and clinical characteristics of severe fever with thrombocytopenia syndrome virus in Gangwon Province, Korea, a highly prevalent region, 2019–2021. Microorganisms 11: 2288. 37764132 10.3390/microorganisms11092288 PMC10536435

[62] Gokuden M , 2018. Low seroprevalence of severe fever with thrombocytopenia syndrome virus antibodies in individuals living in an endemic area in Japan. Jpn J Infect Dis 71: 225–228. 29709983 10.7883/yoken.JJID.2017.497

[63] Matsumoto C Shinohara N Furuta R Tanishige N Shimojima M Matsubayashi K Nagai T Tsubaki K Satake M, 2018. Investigation of antibody to severe fever with thrombocytopenia syndrome virus (SFTSV) in blood samples donated in a SFTS‐endemic area in Japan. Vox Sang 113: 297–299. 29359332 10.1111/vox.12629

[64] Kuba Y , 2021. Seroepidemiological study of severe fever with thrombocytopenia syndrome in animals and humans in Okinawa, Japan. Ticks Tick Borne Dis 12: 101821. 34525434 10.1016/j.ttbdis.2021.101821

[65] Ando T , 2021. Severe fever with thrombocytopenia syndrome in cats and its prevalence among veterinarian staff members in Nagasaki, Japan. Viruses 13: 1142. 34198717 10.3390/v13061142 PMC8232257

[66] Kirino Y , 2021. Seroprevalence of severe fever with thrombocytopenia syndrome virus in small-animal veterinarians and nurses in the Japanese prefecture with the highest case load. Viruses 13: 229. 33540629 10.3390/v13020229 PMC7912989

[67] Hidaka K Mitoma S Norimine J Shimojima M Kuroda Y Hinoura T, 2024. Seroprevalence for severe fever with thrombocytopenia syndrome virus among the residents of Miyazaki, Japan: An epidemiological study. J Infect Chemother 30: 481–487. 38042299 10.1016/j.jiac.2023.11.026

[68] Kato H Yamagishi T Shimada T Matsui T Shimojima M Saijo M Oishi K; SFTS epidemiological research group—Japan, 2016. Epidemiological and clinical features of severe fever with thrombocytopenia syndrome in Japan, 2013–2014. PLoS One 11: e0165207. 27776187 10.1371/journal.pone.0165207 PMC5077122

[69] Kobayashi Y ; SFTS Epidemiological Research Group Japan, 2020. Severe fever with thrombocytopenia syndrome, Japan, 2013–2017. Emerg Infect Dis 26: 692–699. 32186502 10.3201/eid2604.191011 PMC7101122

[70] Dong Y Lin SH Jiang L Liu H, 2022. Clinical characteristics and risk factors of 267 patients having severe fever with thrombocytopenia syndrome-new epidemiological characteristics of fever with thrombocytopenia syndrome: Epidemiological characteristics of SFTS. *Medicine (Baltimore)* 101: e31947. 36550925 10.1097/MD.0000000000031947 PMC9771163

[71] Gao S Geng X Lu Q Wu S Shan Z Chang C, 2023. Epidemiological characteristics and spatio-temporal aggregation of severe fever with thrombocytopenia syndrome in Jinan City, China, 2018–2022. PLoS Negl Trop Dis 17: e0011807. 38134002 10.1371/journal.pntd.0011807 PMC10745217

[72] Sun J , 2014. Epidemiological characteristics of severe fever with thrombocytopenia syndrome in Zhejiang Province, China. Int J Infect Dis 25: 180–185. 24947422 10.1016/j.ijid.2014.02.022

[73] Zhan J Cheng J Hu B Li J Pan R Yang Z Zhou W Zhan F Gou D, 2017. Pathogens and epidemiologic feature of severe fever with thrombocytopenia syndrome in Hubei province, China. Virus Res 232: 63–68. 28089865 10.1016/j.virusres.2017.01.009 PMC7114523

[74] Tao M , 2021. Severe fever with thrombocytopenia syndrome in southeastern China, 2011–2019. Front Public Health 9: 803660. 35223761 10.3389/fpubh.2021.803660 PMC8864090

[75] Wang W Zhang A Wu Q Zhu L Yang J, 2022. Epidemiological and clinical characteristics of severe fever with thrombocytopenia syndrome in southern Anhui Province, China, 2011–2020. Jpn J Infect Dis 75: 133–139. 34470972 10.7883/yoken.JJID.2021.391

[76] Zhang Y Miao W Xu Y Huang Y, 2021. Severe fever with thrombocytopenia syndrome in Hefei: Clinical features, risk factors, and ribavirin therapeutic efficacy. J Med Virol 93: 3516–3523. 32965706 10.1002/jmv.26544

[77] Ding F , 2014. Risk factors for bunyavirus-associated severe fever with thrombocytopenia syndrome, China. PLoS Negl Trop Dis 8: e3267. 25330383 10.1371/journal.pntd.0003267 PMC4199554

[78] Hu JL , 2016. Risk factors for bunyavirus-associated severe fever with thrombocytopenia syndrome: A community-based case-control study. PLoS One 11: e0166611. 27846273 10.1371/journal.pone.0166611 PMC5112944

[79] Sun J , 2016. Factors associated with severe fever with thrombocytopenia syndrome infection and fatal outcome. Sci Rep 6: 33175. 27605309 10.1038/srep33175 PMC5015071

[80] Wen Y Fang Y Cao F Zhang G Cheng S Yu Y Huang R Ni Z Li J, 2024. A person-to-person transmission cluster of severe fever with thrombocytopenia syndrome characterized by mixed viral infections with familial and nosocomial clustering. Heliyon 10: e24502. 38298613 10.1016/j.heliyon.2024.e24502 PMC10827760

[81] Hu L , 2022. Predisposing factors for person-to-person transmission of severe fever with thrombocytopenia syndrome bunyavirus. J Hosp Infect 123: 174–178. 34767872 10.1016/j.jhin.2021.10.023

[82] Yoo JR Choi JH Kim YR Lee KH Heo ST, 2019. Occupational risk of severe fever with thrombocytopenia syndrome in healthcare workers. Open Forum Infect Dis 6: ofz210. 31139678 10.1093/ofid/ofz210 PMC6527088

[83] Qian J Wei J Ren L Liu Y Feng L, 2023. Sex differences in incidence and fatality of severe fever with thrombocytopenia syndrome: A comparative study based on national surveillance data of China. J Med Virol 95: e28632. 36866702 10.1002/jmv.28632

[84] Cui H , 2024. Global epidemiology of severe fever with thrombocytopenia syndrome virus in human and animals: A systematic review and meta-analysis. Lancet Reg Health West Pac 48: 10113. 10.1016/j.lanwpc.2024.101133 PMC11261768 39040038

