# Supplementary material for: Epidemiological Characteristics of Severe Fever with Thrombocytopenia Syndrome
Source: Am J Trop Med Hyg. 2025 Feb 18;112(5):956–62. doi: 10.4269/ajtmh.24-0616 (PMC12062676; doi:10.4269/ajtmh.24-0616)
Supplement: Supplemental Materials [file tpmd240616.SD1.pdf]

Supplemental Table 1
Search terms and strategy (as of 14 February 2024)

**Database**	**Search Term**	**Results**
PubMed	#1 “Severe fever with thrombocytopenia syndrome”	1,088
	#2 SFTS	1,508
	#3 SFTSV	656
	#4 “Dabie bandavirus”	54
	#5 Epidemiology	3,141,320
	#6 Incidence	3,793,684
	#7 Prevalence	3,666,911
	#8 Seroprevalence	38,418
	#9 Seropositive	66,351
	#10 “Risk factor*”	1,417,438
	#11 #1 OR #2 OR #3 OR #4	1,832
	#12 #5 OR #6 OR #7 OR #8 OR #9 OR #10	4,921,889
	#13 #11 AND #12	616
Scopus	#1 “Severe fever with thrombocytopenia syndrome”	2,958
	#2 SFTS	2,527
	#3 SFTSV	1,061
	#4 “Dabie bandavirus”	85
	#5 Epidemiology	5,030,764
	#6 Incidence	4,382,937
	#7 Prevalence	4,362,135
	#8 Seroprevalence	127,758
	#9 Seropositive	112,356
	#10 “Risk factor*”	4,310,504
	#11 #1 OR #2 OR #3 OR #4	4,559
	#12 #5 OR #6 OR #7 OR #8 OR #9 OR #10	11,103,828
	#13 #11 AND #12	2,309

Supplemental Table 2
Summary of epidemiological, characteristics of SFTS infection
and SFTS distribution patterns

**Author, Year**	**Study Period**	**Study Site(s)**	**Study Population /Samples**	**Epidemiology**	**Characteristics of Infection and Distribution Pattern**
Cui, 2013 [11]	2008-2010	Yiyuan County, Shandong, China	Blood samples of patients diagnosed as anaplasmosis (n=21) and healthy volunteers	Seroprevalence: 1.3%	Median age was 67 years, and most were farmers.

			in the villages (n=78)		
Ding, 2014 [50]	2010-2011	Laizhou County, Shandong, China	Healthy volunteers (n=1,060)	Incidence: $4.1/10^5$ population	Median age was 59 years. Incidence was higher in patients over 40 years old.
Liang, 2014 [25]	2011	Jiangsu, China	General population of studied area (n=2,510)	Seroprevalence: 0.44%	Farming, raising goats and taking animals to graze were risk factors for SFTSV infection.
Ding, 2014 [76]	2011	Henan, Hubei and Shandong, China	SFTS cases reported to Chinese Center for Disease Control and Prevention (n=134) and randomly selected controls (n=288)	N/A	Tick bites 2 weeks before onset, owning cattle, owning cats, presence of ticks in living area, working in the field and presence of weeds and shrubs in working areas associated with SFTS.
Liu, 2014 [45]	2011-2012	Xinyang City, Henan, China	SFTS cases (n=504)	N/A	Annual peak of SFTS was around May to July. Median age was 61 years old. Annual incidence increased with age. Most cases were females. SFTS incidence was associated with shrubs, forests, and croplands.
Sun, 2014 [71]	2011-2013	Zhejiang, China	SFTS cases (n=65)	N/A	Median age was 66 years. Most

					cases occurred in persons aged 50 years and above. Most cases occurred during May and August. Common exposure history included doing outdoor activities, exposed to tick, bred domestic animals, and had history of exposure to a mouse.
Li, 2014 [33]	2012-2013	Jiangsu, China	Farmers (n=2,547)	Seroprevalence: 1.3%	Mean age of seropositive farmers was 56.5 years. Seroprevalence increased with age. Seropositive was more prevalent in males than females.
Zhang, 2014 [35]	2013	Pujiang district, Shanghai, China	Healthy volunteers in studied area (n=54)	Seroprevalence: 7.4%	N/A
Liu, 2015 [17]	2010-2013	China	Laboratory -confirmed SFTS cases (n=1,786)	N/A	Annual cases increased significantly. Most cases occurred during May to July. Median age was 61 years. Incidence increased with age. Most cases were farmers and forest workers living in rural

					areas and doing agricultural activities.
Hu, 2015 [36]	2011-2013	Xinyang City, Henan, China	People living and working in Xinyang who visited a local hospital (n=5,245)	Seroprevalence: 6.59%	Most cases were reported from May to August. Highest seropositivity was found in subjects aged 60 years and above. Seroprevalence was higher in those living in rural areas and those sampled in post-pandemic seasons.
Zeng, 2015 [34]	2012	Henan and Sichuan, China	Plasma samples of blood donors (n=17,208)	Seroprevalence: 0.27-0.54%	N/A
Sun, 2015 [24]	2013	Zhejiang, China	People living in Zhejiang province (n=1,380)	Seroprevalence: 5.51%	Seroprevalence in people age >70 years and >1 year was higher than in >50 and >55 years group. Seroprevalence in family member or living in the same village of patients were higher than those living in different village. Seroprevalence in participants who bred domestic animals were higher than those who did not.

Hu, 2016 [77]	2011-2013	Jiangsu, China	SFTS cases (n=69) and matched controls (n=265)	N/A	Median age of cases was 59.5 years. Most of the cases were farmers (87%). Tick bites 2 weeks before disease onset and presence of weeds and shrubs around the house were risk factors.
Xing, 2016 [39]	2012-2013	Hubei, China	Village residents (n=419)	Seroprevalence: 8.4%	Tick bites were associated with SFTS infection.
Kato, 2016 [67]	2013-2014	Japan	SFTS cases notified to national surveillance system (n=96)	N/A	Most of the cases occurred during April and August. Median age was 78 years. Most cases were retired, unemployed or farmers.
Choi, 2016 [59]	2013-2015	South Korea	SFTS cases reported to Korea Centers for Disease Control and Prevention (n=172)	Incidence: $0.11/10^5$ population overall. Highest incidence in Jeju ($1.26/10^5$ population)	Most cases occurred during May to October. Hilly areas in the eastern and southeastern and Jeju Island was high incidence. Annual incidence has increased through the studied period.
Park, 2016 [58]	2013-2015	South Korea	Hospitalized patients with symptoms like SFTS (n=1,697)	N/A	Majority of cases were older patients. Cases occurred between April to November, with peaks in July and

					October. Most cases occurred in southeast part of the country.
Lyu, 2016 [23]	2014	Anhui, China	Residents of villages with highest rates of endemic SFTS infection (n=2,126)	Seroprevalence: 4.66%	Living in areas of uncontrolled vegetation growth, long-term residents of locality and history of tick bites were risks of infection.
Sun, 2016 [78]	2015	China	SFTS cases (n=72) and matched controls (n=144)	N/A	Breeding domestic animals and history of tick bites were associated with SFTS.
Xing, 2017 [38]	2011	Hubei, China	SFTS cases (n=68) and matched controls (n=136) living in the studied area	N/A	Contact with cattle tick was the risk of SFTS infection.
Zhan, 2017 [72]	2011	Hubei, China	SFTS patients (n=241)	Incidence: $0.42/10^5$ population	All cases occurred during April to December, with peak from April to August. Most were patients aged 50-90 years (median at 55 years). Incidence increased with age. Most of confirmed cases were in the Dabie mountains area.
Wang, 2017 [40]	2011-2016	Hubei, China	Laboratory -confirmed	N/A	Most cases were female and farmer. Mean

			SFTS cases (n=521)		age was 59.3 years. Most common duration of infection was from May to July.
Sun, 2017 [41]	2011-2016	China	SFTS cases reported to China Information System for Disease Control and Prevention (n=5,360)	N/A	Annual cases increased year by year. Females accounted for more cases than males. Most were farmers. Numbers of affected counties increased year by year. Cases occurred from April to October with peaks in May, June, and July.
Huang, 2017 [27]	2012	Yixian County, Anhui, China	Residents in the studied area (n=270)	Seroprevalence: 6.3%	There was significant difference in seropositivity between tea pluckers and non-tea pluckers. Increasing seropositivity was found with age. Men had more seropositive rate than women.
Tian, 2017 [28]	2013	Shaanxi, China	Residents in the studied area (n=936)	Seroprevalence: 4.7%	N/A
Kim, 2017 [51]	2015	Busan, South Korea	Leftover serum of hospital	Seroprevalence: 2.1%	Seroprevalence increased with age. All seropositive

			patients (n=1,069)		cases were from Busan and Gyeongnam. Most were from rural areas.
Kim, 2018 [53]	2010	Jeju, South Korea	Serum of admitted patients who had high erythrocyte sedimentation rates (ESRs) (n=58)	Seroprevalence: 3.4%	N/A
Li, 2018 [29]	2010-2017	Shandong, China	Healthy persons (n=1,375)	Seroprevalence: 3.85% by ELISA and 0.58% by neutralizing antibody	N/A
Han, 2018 [52]	2013-2014	Rural areas in Chungcheongnam-do Province, Jeollanam-do Province and Gyeosangnam-do Province, South Korea	Residents of the studied area (n=1,228)	Seroprevalence: 4.1%	Seroprevalence was significantly higher in people aged 65 years and older, living for a long duration in the area, long career duration (raising animals and livestock) and had history of fever and gastrointestinal symptoms in the past 3 years.
Gokuden, 2018 [61]	2015	Kagoshima, Japan	Residents of studied area (n=646) and serum samples collected from blood donors Japanese	Seroprevalence: 0.3% from residents and 0% from serum samples	N/A

			Red Cross Kyushu Block Blood Center (n=1,000)		
Matsumoto, 2018 [62]	2015-2016	Chugoku-Shikoku, Japan	Donated blood samples from the studied area (n=3,990)	Seroprevalence: 0%	N/A
Kimura, 2018 [8]	Up to 2017	Ehime, Japan	People age of 50 years and over (n=694)	Prevalence: 0.14%	N/A
Yoo, 2019 [56]	2015-2017	Jeju, South Korea	Farmers (n=254)	Seroprevalence: 2.4%	Fruit farmers were at higher risk than other farmers.
Du, 2019 [31]	2016	Henan, China	Healthy people in the studied area (n=1,463)	Seroprevalence: 10.46% for IgG and 0.82% for IgM	N/A
Shen, 2019 [26]	2018	Dachen Island, Zhejiang, China	Inhabitants of Dachen Island (n=439)	Seroprevalence: 3.0% for total antibodies and 0.5% for IgM	All seropositive cases were reported in people aged >50 years.
Yoo, 2019 [81]	2018	Jeju, South Korea	Secondary patient contracted SFTS as a healthcare-associated infection from index patient (n=9)	N/A	Direct contact with index case, such as living in the same household and performing medical procedure without wearing mask, can results in seropositivity.
Kobayashi, 2020 [68]	2013-2017	Japan	SFTS patient data from Japan's	N/A	Contact with companion animal, history of tick bite,

			National Epidemiologic Surveillance of Infectious Disease (n=117)		contact with saliva of symptomatic cat.
Zohaib, 2020 [16]	2016-2017	Khyber Pakhtunkhwa, Punjab, Sindh and Balochistan, Pakistan	Farmers of livestock (n=1,657)	Seroprevalence: 46.7% for ELISA-based test (unspecific to SFTS) and 2.5% for microneutralizing test (specific to SFTS)	From ELISA-based test, the seroprevalence increased with age and was higher in females than in males.
Win, 2020 [13]	2018-2019	Sagaing and Magway, Myanmar	Blood samples from clinically suspected scrub typhus patients (n=152)	N/A	Co-infection between scrub typhus and SFTSV was found in 3.3% of samples. All SFTS cases had no travel history and lived in a village in Sagaing.
Fang, 2021 [18]	1996-2019	China and South Korea	27 clusters of SFTS patients (n=138)	N/A	Cases usually occurred in elderly people in May, June, and October in central and eastern China.
Miao, 2021 [9]	2010-2018	China	SFTS cases reported to China Information System for Disease Control and Prevention (n=7,721)	Incidence $0.064/10^5$ population	Age ≥60 years old, living in rural areas with crop fields and tea farms. Cases were clustered in Changbai Mountain, Jiaodong Peninsula, Taishan

					Mountain, and Huaiyangshan Mountain. Distribution of SFTS was influenced by elevation, woods coverage and near proximity with habitats of migratory birds.
Huang, 2021 [22]	2010-2019	China	SFTS cases, including both lab-confirmed and suspected cases (n=13,824)	N/A	Most cases were middle-aged and elderly people. Farmers were main high-risk group. SFTS has an increasing incidence and geographical distribution. Most cases occurred during April to October.
You, 2021 [30]	2011-2018	Anhui, China	Healthy residents in the studied area (n=754)	Seroprevalence: 20.16%	Living in hilly areas, direct contact with domestic livestock and knowing that tick bite can spread the disease were risk factors of SFTS.
Tao, 2021 [73]	2011-2019	Zhejiang, China	SFTS patients (n=463)	N/A	Elderly farmers accounted for most cases. Most cases occurred during April and August.
Kuba, 2021 [63]	2015-2017	Okinawa, Japan	Healthy person (n=537)	Seroprevalence: 0%	N/A
Noh, 2021 [54]	2017-2018	South Korea	Forest and field	Seroprevalence: 0.2%	N/A

			workers (n=583)		
Ye, 2021 [37]	2018	Xinyang City, Henan, China	Healthy residents of studied area (n=886)	Seroprevalence: 11.9%-15.8% for IgG and 6.8%-9.8% for neutralizing antibody.	Risks of seropositivity included aged ≥70, contact with cats and working in tea garden.
Kirino, 2021 [65]	2018	Miyazaki, Japan	Small-animal veterinarians and nurses (n=90) and serum samples from Japanese Red Cross Society (n=1,000)	Seroprevalence: 2.2% in veterinarians and nurses and 0% in healthy blood donors	N/A
Ando, 2021[64]	2018-2020	Nagasaki, Japan	Veterinarians and nurses in animal hospitals (n=71)	Seroprevalence: 4.2%	N/A
Ye, 2021 [6]	Up to 2020	China	Secondary SFTS cases in 23 clusters (n=89)	N/A	Risks of human-to-human transmission included exposure to index cases' blood.
Zhang, 2021 [75]	2020	Anhui, China	SFTS cases (n=62) and matched controls (n=124)	N/A	Risk factors for SFTS infection were farmer, tick bite history and having weeds and shrubs in working areas.
Chen, 2022 [44]	2010-2017	Shandong, China	SFTS cases (n=2,731)	Incidence: 0.59-6.32/10^5 population	Cases were seasonal, with high incidence during May and October. Most

					cases occurred in individuals aged over 40 years, with highest peak in 60-65 age group. The numbers of affected cities increased from 5 in 2010 to 14 in 2017.
Wang, 2022 [47]	2011-2019	Liaoning, China	SFTS cases (n=783)	Incidence: $0.2/10^5$ population	Males accounted for more cases than females. Cases were concentrated in 60-69 age group. Incidence showed upward trend and seasonal characteristics. Reported cases mainly occurred between May and October.
Zu, 2022 [42]	2011-2020	Zhejiang, China	Hospitalized SFTS cases reported to Taizhou Center for Disease Control and Prevention (n=188)	N/A	Annual incidence of SFTS increased, as well as epidemic area. Most cases occurred in hilly areas and coastal areas. Majority of cases were 60 years and older.
Wang, 2022 [74]	2011-2015 and 2016-2020	Anhui, China	SFTS cases (n=86)	N/A	Farmers are the high-risk group of SFTS. Most cases occurred from May to July.
Chen, 2022 [21]	2011-2021	China	Cases reports regarding	N/A	Number of clusters increased from

			35 SFTS clusters reported to Public Health Emergency Information Management System of China Information System for Disease Control and Prevention (n=118)		April to September. Contact with blood or bloody fluids was the risk for human-to-human transmission.
Kim-Jeon, 2022 [55]	2014	Mui Island, Incheon, South Korea	Residents in the studied area (n=203)	Seroprevalence: 5.9%	Seropositive rate was higher in age more than 65 years than younger people.
Wang, 2022 [46]	2014-2018	Shandong, China	SFTS cases reported to China Information System for Disease Control and Prevention (n=2,814)	Incidence: $0.57/10^5$ population	Distribution of SFTS was in clustering pattern.
Wang, 2022 [48]	2016-2018	Shandong, China	SFTS cases (n=2,814)	Incidence: $0.627/10^5$ population	N/A
Dong, 2022 [69]	2016-2020	Weihai City, Shandong, China	SFTS patients (n=846)	N/A	72.41% had history of fieldwork, 21.27% had history of tick bite. Annual peak season was from April to November.

Tran, 2022 [14]	2017-2018	Thua Thien Hue and Quang Nam, Vietnam	Healthy residents in the area, mostly were farmers (n=714)	Seroprevalence: 3.36% for IgM and 3.64% for IgG	N/A
Rattanakomol, 2022 [15]	2019-2020	Bangkok, Thailand	Serum samples of patients with acute fever (n=712)	Seroprevalence: 0.42%	N/A
Hu, 2022 [80]	2020	Anhui, China	Secondary SFTS cases (n=7)	N/A	Nosocomial person-to-person transmission was reported. High viral load, exposed to bloody secretions and bleeding, exposure time ≥30 min to index patient and face-to-face contact distance ≤50 cm was risks of person-to-person transmission.
Qian, 2023 [82]	2010-2018	China	SFTS cases reported to China Information System for Disease Control and Prevention (n=7,724)	N/A	Females had more significant incidence rate than males. Significant difference was observed in people aged 40-69 and 60-69 years.
Ding, 2023 [49]	2010-2019	China	Laboratory-confirmed SFTS cases from China Information System	Incidence: 0.1 to 0.3/10^5 population	Most cases occurred during April to September. By 2089, incidence of SFTS is

			for Disease Control and Prevention (n=8,902)		projected to be increased due to broader distribution of *H. longicornis*, especially in northern and northwestern China.
Hou, 2023 [12]	2010-2019	Yantai City, Shandong, China	Laboratory-confirmed SFTS cases (n=968)	N/A	Presence of rats in household, history of tick bite before onset and presence of weeds and shrubs around the house were risks of SFTS. Most cases occurred during May to August.
Liu, 2023 [20]	2011-2020	Henan, China	SFTS cases (n=1,219)	N/A	Median age was 64 years old. Most patients aged 50 years and older and were female. Higher SFTS incidence was observed in villages with high plant coverage, tea plantation and goat density.
Liang, 2023 [19]	2011-2021	Jiangsu, China	SFTS cases (n=538)	Incidence: 002 to $0.14/10^5$ population	Elderly farmers accounted for most cases. Most cases occurred from May to August. Cases were clustered in western region of Jiangsu province

Hidaka, 2023 [66]	2014-2019	Miyazaki, Japan	Residents of studied area (n=6,013)	Seroprevalence: 0.9%	Age 60-75 years and farmers were risk factors of seropositivity.
Zhang, 2023 [43]	2015-2021	Hefei City, Anhui, China	SFTS cases reported to China Information System for Disease Control and Prevention (n=370)	Incidence: $0.65/10^5$ population	Incidence rate increased yearly.
Gao, 2023 [70]	2018-2022	Jinan City, Shandong, China	SFTS cases reported to China Information System for Disease Control and Prevention (n=680)	N/A	Most of cases occurred between April and October, older than 50 years and were farmers.
Moon, 2023 [60]	2019-2021	Gangwon, South Korea	Patients with suspected SFTS (n=776)	Incidence: $1.34/10^5$ population	Most were females. Median age was 67 years. Highest frequency was in 60-69 years group. Most cases occurred during June and October.
Kim, 2023 [57]	2021	Gwangju, South Korea	Veterinarian hospital staffs (n=103)	Seroprevalence: 3.9% for ELISA, 2.9% for immunofluorescence assay and 1.9% for 50% plaque reduction neutralization antibody test	Participants who lacked awareness about animal-to-human transmission had significantly more seropositivity than those who did not.
Zu, 2024 [32]	2020-2021	Zhejiang, China	People living in	Seroprevalence: 4.8% for total	Living on island was associated

			the area (n=862)	antibody and 0.6% for IgM	with SFTS seropositivity.
Wen, 2024 [79]	2023	Zhejiang, China	A cluster of SFTS cases (n=6)	N/A	Exposed to serum and blood of index case can transmitted SFTSV.

N/A = not applicable
